# Supplementary material for: Patterns of Intron Gain and Loss in Fungi
Source: PLoS Biol. 2004 Nov 30;2(12):e422. doi: 10.1371/journal.pbio.0020422 (PMC532390; doi:10.1371/journal.pbio.0020422)
Supplement: Table S1 — Also available at http://genes.mit.edu/NielsenEtAl/. (4.3 MB ZIP). [file pbio.0020422.st001.zip › NielsenEtAl/html/732.html]

AN2002.1.NCU05999.1.MG01287.1.FG08771.1


```
 CLUSTAL W (1.82) Multiple Sequence Alignments - Introns Inserted


Sequence 1: MG01287.1	492 aa
Sequence 2: FG08771.1	464 aa
Sequence 3: AN2002.1	459 aa
Sequence 4: NCU05999.1	466 aa
Alignment Length: 535 aa
Number Identitical Residues: 158 aa
Alignment Score (without introns) 8923


MG01287.1 	MDSSSSVATSTSSSSNHRLVRSSEGSPSAGGDDIEEVIMTPGIATGRVQPAVSV--AIPD
NCU05999.1	---MAASTTSSFPTSQEGVATSSQQNLDAITDDDFEYVDEEFVLSHSAEQQAQPEPMIPD
FG08771.1 	-------------------MTSVQKQPQPG------VHMEH---------------NIPQ
AN2002.1  	---------------------MADATIHAA---------------------------IPR
          	                       :    .                            ** 

MG01287.1 	LFTQLPPVKDDLATSTSKTQDETVAICLPYLAGSDANADVEHNAHGVPHIDRKKHVRFLR
NCU05999.1	LFKQPSLVKDDLITATSQLQDQTVEHCLPFLAGEDGP--LDLNDYGLPKLNREKHIKFLK
FG08771.1 	LFTTDPPIYDDLPTESSRLQDETINEVLPYLNGTEH---KQCNSHGLPHLDRRRHVNFLH
AN2002.1  	LFQEPPVIRDTLITETSELQAETINKCLPFLAGAHSSQKGPLNRHGVPSLMRDVHIGYLC
          	**   . : * * * :*. * :*:   **:* * .   .   * :*:* : *  *: :* 

MG01287.1 	NMLRQLPAPFIAADASRPWFLYWSLNAMAILGENVKEDYAES~LADTARSMQNESGGFSG
NCU05999.1	QSLGPLPGRFVAVDASRPWYLYWCLSGLTMMGEDVSS-YRDS~VIETARTMQNESGGFGG
FG08771.1 	KQLGKLPAAFTSADPSRPWFFYWCLSALVLLGEDVEP-YRER~LVDTVRPMQNADGGFAG
AN2002.1  	DSLEDYPPGFVAMDASRPWMIYWALAALSLLGEDLSR-HRER2AIATITPMQNPTGGFGG
          	. *   *  * : *.**** :**.* .: ::**::.  : :     *  .***  ***.*

MG01287.1 	GHGQTSHLATTYAVVLALAVVGDEEGLSLIDRRAL~WKWLCDLKEADGGFRMSLGGEEDV
NCU05999.1	GHGQTSHLATTYAVILAIALVGGEEAYDVIDKKAM~WKWLCSLKQPDGGFQVCVGGEEDI
FG08771.1 	GFGHTSHLATSYATVLSLALVGGEDAYECIDRRAM~WRWLCSLKQPDGGFQMALGGEEDV
AN2002.1  	GHGQISHCASSYAAVLSLAMVGGEEAFNLIDRQAM2WKWLGKLKQPDGGITVCEGGEEDV
          	*.*: ** *::**.:*::*:**.*:. . **::*: *:** .**:.***: :. *****:

MG01287.1 	R2GAYCAAVIISLLNLPLDLCKDSEAYIRDPTANLFTGLGDYVRKC1QTFEGGISGQPDA
NCU05999.1	R2GAYIAAVIITLLDLPLDLTPESPAYDG--RSNLLTGLAEYVRSC1QTFEGGISSQPNN
FG08771.1 	R2GAYCAAVIISLLNLPLELSQDSPARSAGHTG-LFAGLANYVHRC1QTHEGGVSAKPGI
AN2002.1  	R2GAYCAMVIISLLNLPLTLPPEAKAREYGLET-FTDGLWEYLSRC1QTFEGGISGSPGS
          	* *** * ***:**:*** *  :: *   .    :  ** :*:  * **.***:*..*. 

MG01287.1 	EAHGAYAFCALGCLSLLGTPSETIPK2YLNIERLISWLSSRQYA~PEGGFSGRTNKLVDG
NCU05999.1	EAHGAYAFCALACLAILDNPRRIIP-~----------------S2P-SGPGAEKNKTATG
FG08771.1 	EAHGAYAFCALGCLSIIDSPHRAIPR2HLNVPLLISWLSSRQYA~PEGGFSGRTNKLVDG
AN2002.1  	EAHGAYAFCALACLCILGEPEATIPK2CMDVQMLLSWLSARQYA~PEGGFAGRTNKLVDG
          	***********.**.::. *   **    .     :  :: . : *..* ....** . *

MG01287.1 	CYSHWVGGCWPLIEACLNGP------VKVSS----LDVEPQPLFSREGLMRYILCCCQEQ
NCU05999.1	-----------------------------------LAAAPESLYSREGLIRYILCCCQDQ
FG08771.1 	CYSHWVGGCWPLIEAALNGPGSGPEDAEASSGGRSLPVAQSSLFSREGLIRYILCCCQDL
AN2002.1  	CYSHWVGSCWPLIQSALDGV------QDAAG----PKPAVGNLYSREGLTRYILGCCQ--
          	. :   ... .  .:. ..        . :.           *:***** **** ***  

MG01287.1 	GKRGGLRDKPGK2PSDAYHSCYVLSGLSSAQN-RWQLVVG-----DDDMP----------
NCU05999.1	TKRGGLRDKPYK2MSDPYHTNYVLSGLSSAQH-QWDLDSDPLPLPNGDTTPSLAALGADS
FG08771.1 	SKRGGLRDKPSK2YSDAYHTCYVLSGLSSAQH-RWTLDTARS--HEADVKG-------D-
AN2002.1  	SKNGGLRDKPGK2HPDSYHTCYTLTGLSSVQHYHYHTNSSVV--PNGRFSS-------AF
          	 *.******* *  .*.**: *.*:****.*: ::          :              

MG01287.1 	AWMVSPFPNEEE------IFDEKDRVGTVHPVYVIPEDKVAKVQTFFASR--DGF-----
NCU05999.1	VWNVFPYIDENDQ-----IYEDSDRVRPIHPAYAIPQKNVNAIRAYFQGR--SGFASTLG
FG08771.1 	SWSVTPYMDGEQ------IFDEEDRVATVHPVYVIPQHKVEGMQNYFSSK--QGF-----
AN2002.1  	SWKYRPAWDSDEKGYDVNVFDESDRLKAFHPLYVIPHKAAENMRLWFEQKPWENVGE---
          	 *   *  : ::.. . .::::.**: ..** *.**.. .  :: :*  :. .....   

MG01287.1 	--
NCU05999.1	GE
FG08771.1 	--
AN2002.1  	--
          	
```
